# Safety, tolerability, pharmacokinetics and pharmacodynamics of belimumab in Japanese patients with mild-to-moderate systemic lupus erythematosus

**DOI:** 10.3109/21556660.2013.792823

**Published:** 2013-04-12

**Authors:** Masanori Yamada, Mikio Akita, Tomofumi Nakagawa, Naoki Takahashi, Akira Endo, Pascal Yoshida

**Affiliations:** 1 Medicines Development, Japan Development & Medical Affairs Division, GlaxoSmithKline K.K., TokyoJapan; 2 Clinical Pharmacology Department, Medicines Development, Japan Development & Medical Affairs Division, GlaxoSmithKline K.K., TokyoJapan; 3 Biomedical Data Sciences Department, Japan Development & Medical Affairs Division, GlaxoSmithKline K.K., TokyoJapan; 4 Clinical Safety & PMS, Japan Development & Medical Affairs Division, GlaxoSmithKline K.K., TokyoJapan

**Keywords:** Belimumab, Safety, Pharmacokinetics, Pharmacodynamics, Systemic lupus erythematosus

## Abstract

**Objectives:**

Belimumab, an anti-B lymphocyte stimulator (BLyS) human monoclonal antibody, was approved in the United States, Canada and European Union for the treatment of the patients with systemic lupus erythematosus (SLE). However, belimumab had not been evaluated in Japanese patients. The objectives of this study were to evaluate the safety and tolerability of belimumab in Japanese patients with SLE, as well as to investigate the pharmacokinetics (PK) and biological activity of belimumab in this population.

**Methods:**

A total of 12 Japanese patients were enrolled in a randomized, single-blind, placebo-controlled, dose-ascending design study. A dosing regimen of a single intravenous infusion over 1 hour of belimumab (1 mg/kg and 10 mg/kg) was employed. Patients were followed for 84 days after dosing to assess adverse events, pharmacokinetics, biomarkers and SLE disease activity.

**Clinical trial registration number:**

ClinicalTrials.gov identifier is NCT01381536.

**Results:**

Belimumab (1 mg/kg and 10 mg/kg) demonstrated a favorable clinical safety and tolerability profile in Japanese patients with SLE. The incidence of adverse events was similar among the two belimumab groups and placebo group. The PK profile of single-dose belimumab was approximately dose proportional, and the long terminal elimination half-life (12.4–15.7 days), low clearance (3.55–4.65 mL/day/kg), and small volume of distribution (76.2–80.1 mL/kg) were consistent with a fully humanized antibody. Effects of belimumab on B cells suggested biological activity effects expected as an inhibitor of BLyS.

**Limitation:**

The small sample size and single dose design of this study prevent definitive conclusions regarding the safety, pharmacokinetics or pharmacodynamics of belimumab in a Japanese population being made.

**Conclusions:**

The preliminary safety, PK profile, and observed biological activity of belimumab support further evaluation of its safety and efficacy in Japanese patient with SLE.

## Introduction

Systemic lupus erythematosus (SLE) is a chronic autoimmune disorder characterized by autoantibody production and abnormal B lymphocyte function1. Although high-dose corticosteroids and immunosuppressants are widely used for treatment, some patients with SLE are refractory to these conventional treatments. In addition to the background of considerable progress in conventional therapies, the United States Food and Drug Administration (FDA) and the European Medicines Evaluation Agency (EMEA) approved belimumab as a new biological agent for the treatment of SLE in 2011.

Belimumab is a recombinant, human, immunoglobulin G1 lambda (IgG1λ) monoclonal antibody that binds soluble B lymphocyte stimulator (BLyS) protein with high affinity and inhibits its biological activity2. BLyS is overexpressed in patients with SLE and correlates with severities in disease activity. Approval of belimumab was based on two clinical studies, BLISS-52 and BLISS-76, both of which demonstrated benefit when belimumab was superimposed on standard-of-care3–5. Based on positive results in efficacy in both studies, as well as a favorable safety profile, belimumab was approved for patients with many of the diverse manifestations of lupus. However, since patients with active nephritis and central nervous system involvement were excluded from the studies, belimumab is not approved for these indications. Data in Asian populations was also limited, and Japanese patients were not included in either pivotal study.

Clinically significant inter-ethnic differences in drug response and pharmacokinetics have been reported for a number of drugs6,7. Differences in drug response may be attributable to differences in average pharmacokinetics and pharmacodynamics and in turn variations in drug safety and efficacy between populations have been reported. Additionally, differences across populations in the nature of some diseases could contribute to inter-ethnic differences in drug response.

In the first study of belimumab, SLE patients with stable disease were given various doses of belimumab to study the *in vivo* safety of the drug as well as its pharmacokinetics and pharmacodynamics8. The results of the study concluded that belimumab had a good safety profile *in vivo*, predictable linear pharmacokinetics over a dosage range from 1 to 20 mg/kg, small volume of distribution, slow clearance and a half-life ranging from 8 to 14 days. And belimumab was effective in reducing peripheral B-cell counts. However Japanese patients with SLE have not been included in previous study.

Therefore, as for all pharmacotherapies, an assessment of the potential for ethnic differences is relevant for belimumab. In the present study, Japanese patients with SLE were enrolled, and the primary objective was to evaluate the safety and tolerability of belimumab in Japanese patients. In addition, we examined the pharmacokinetics (PK), and pharmacodynamics (PD) of belimumab in these patients.

## Methods

### Patients

Patients aged 20 years or older with SLE, as defined by the American College of Rheumatology criteria9, were enrolled in this study. All patients were required to be born in Japan with four ethnic Japanese grandparents and possess Japanese citizenship. Eligible patients had stable SLE disease activity, as judged clinically by the principal investigator, for at least 2 months before screening and either maintained with no medication or with a stable SLE treatment. Patients were also positive for anti-nuclear antibody (ANA) or anti-double stranded deoxyribonucleic acid (dsDNA) antibody. Patients with severe active lupus nephritis requiring hemodialysis, cyclophosphamide, or high-dose (>60 mg) prednisone, or who had received intravenous immunoglobulin (IVIG), or plasmapheresis within 6 months of screening were not eligible. Patients with active central nervous system lupus within 6 months of screening, a history of renal transplant, hypogammaglobulinemia or IgA deficiency (IgA <10 mg/dL), evidence of clinically significant non-SLE-related acute or chronic disease, a history of any serious infection within 4 weeks of screening, or a history of an anaphylactic reaction to monoclonal antibodies were excluded. Patients were also excluded if they were tested positive for hepatitis (B or C) or human immunodeficiency virus, or had a history of drug or alcohol abuse. Pregnant or nursing patients were ineligible for inclusion in the study, and adequate practice of contraception was required for participating patients. All patients provided written, informed consent to participate in the study. The institutional review board provided formal approval for the study, which was conducted in accordance with good clinical practice, all known regulatory requirements and the Declaration of Helsinki (as revised in Edinburgh 2000, Washington 2002, and Tokyo 2004).

### Study design

This was a randomized, single-blind, placebo-controlled, dose-ascending design study of single intravenous (IV) doses of belimumab in Japanese patients with SLE. Patients in Cohort 1 received a single administration of placebo or belimumab at a dose of 1 mg/kg. Dosing in Cohort 2 was started after dosing was completed and the safety and tolerability results had been assessed in Cohort 1. Patients in Cohort 2 received a single administration of placebo or belimumab at a dose of 10 mg/kg. Each cohort comprised six patients, and the ratio of patients randomized to receive belimumab versus placebo in each cohort was 2:1.

### Study drug

Belimumab was supplied as a sterile, single-use, lyophilized cake in 20 mL vials containing 400 mg of belimumab plus excipients (citric acid/sodium citrate/sucrose/polysorbate). Upon reconstitution with 4.8 mL of sterile water for injection (SWFI) each vial contained 80 mg/mL of belimumab. Placebo was supplied as sterile, single-use, lyophilized product in 20 mL vials containing same excipients to active vial. All investigational products were stored refrigerated at 2–8°C.

The calculated dose of investigational product to be administered to each patient was determined in milligrams (mg) by the assigned treatment group and the patient's body weight in kilograms (kg) at randomization. The reconstituted investigational product was diluted in 250 mL normal saline for IV infusion. An amount of normal saline, equal to the calculated amount of product to be added was removed from the infusion bag prior to adding the product. After adding the reconstituted product, the bag was gently inverted to mix the solution. Each selected patient was to receive an IV administration of belimumab or placebo over a 1 hour infusion period.

### Safety

Safety profile evaluation included collection of adverse events (AEs) and serious AEs (SAEs), clinical laboratory values, vital signs, and 12-lead ECG from randomization through study day 84. AEs were coded on the basis of the Medical Dictionary for Regulatory Activities terminology (MedDRA). AEs and SAEs were considered treatment emergent if they occurred within 84 days after the dose of study agent. Hematology, clinical chemistry, and urinalysis panels were assessed pre-dose, and at 24 hours, and days 7, 14, 28, 42, 56 and 84 post-dose. Vital signs including systolic and diastolic blood pressure, heart rate, and body temperature were taken before dosing, and at 5 minutes, 1, 6 and 24 hours, and days 2, 7, 14, 21, 28, 42, 56 and 84 post-dose. 12-Lead ECG was evaluated pre-dose, and at 5 minutes, 24 hours and days 14, 28, 56 and 84 post-dose.

Safety data were summarized and listed according to the treatment which was actually taken by each subject. No statistical analysis was performed to compare the safety data between dose groups.

### Pharmacokinetics

Blood samples were collected and analyzed for belimumab pre-dose, and at 5 minutes, 1, 6 and 24 hours, and days 2, 7, 14, 21, 28, 42, 56 and 84 post-dose. Serum concentrations of belimumab were determined using an electrochemiluminescence (ECL)-based assay. Serum concentration–time data for belimumab was analyzed using a compartmental method with the WinNonlin Professional (version 4.1; Pharsight Corporation). The following PK parameters were determined: maximum observed serum concentration (*C*_max_), *C*_max_/dose, area under the serum concentration-time curve from time zero (pre-dose) extrapolated to infinite time (AUC_0–∞_), AUC_0–∞_/dose, distribution phase half-life (*t*_½,α_), terminal phase half-life (*t*_½,β_), systemic clearance of parent drug (CL), volume of distribution for the central compartment (*V*_1_), volume of distribution at steady state (*V*_ss_) and mean residence time (MRT).

### Statistical analysis for PK

An analysis of variance (ANOVA) approach was performed to compare the PK parameters between belimumab dose groups. The dose normalized and log-e transformed *C*_max_ and AUC_0–∞_ were analyzed to estimate the ratio of dose normalized geometric means. An ANOVA model including dose as a fixed effect was fitted to the dose normalized and log-e transformed PK parameters. The dose difference and the corresponding 90% confidence interval based on the ANOVA model were back-transformed to provide the estimate of ratio of dose normalized geometric means.

### Biomarkers

Blood samples were collected and analyzed for biomarkers pre-dose, and days 14, 28, 42, 56 and 84 post-dose. The raw data were summarized for total serum immunoglobulin (IgG, IgM and IgA), autoantibodies (anti-dsDNA antibody, ANA), complement (C3, C4) and CH50, B-cell subsets (CD20+, CD20+/27+ memory, CD20+/27− naïve, CD20+/69+ activated, CD20+/138+ plasmacytoid, CD19+/27^BRIGHT^/38^BRIGHT^ SLE subset and CD20−/138+ plasma cells) and BLyS protein. The percent change from baseline in biomarker was summarized by treatment group at each planned time. No statistical analysis was performed to compare the biomarker data between dose groups.

### Immunogenicity

Blood samples were evaluated for anti-belimumab antibodies at pre-dose, and days 14 and 84. Samples were tested for immunogenicity using an electrochemiluminescence (ECL)-based bridging assay.

These data were summarized and listed according to the treatment which was actually taken by each subject.

## Results

### Patient disposition

Twelve patients were enrolled and assigned to placebo, belimumab 1 mg/kg or 10 mg/kg treatment groups (Table 1). Most patients in the study were female (92%). All patients were of Asian–Japanese heritage with median age of 46.5 years, 42.0 years and 37.0 years in the placebo, belimumab 1 mg/kg and 10 mg/kg groups, respectively. The median SLE disease duration was longer in the placebo group (15.50 years) compared to the belimumab 1 mg/kg group (7.30 years) and belimumab 10 mg/kg group (3.56 years). Patients had mildly to moderately active SLE, which were categorized by SELENA SLEDAI scores ranging from 0 to 8 at study entry. The majority of patients had disease manifestations that included ANA positivity (100%), malar rash (83%), or immunologic disorder (83%) at the time of diagnosis.

**Table 1.** Patient demographics and disease characteristics by treatment groups.

		Belimumab	
	Placebo (*n* = 4)	1 mg/kg (*n* = 4)	10 mg/kg (*n* = 4)
Age, years			
Mean (SD)	45.3 (15.63)	39.3 (10.72)	41.3 (21.09)
Median (range)	46.5 (25–63)	42.0 (24–49)	37.0 (24–67)
Sex, *n* (%)			
Female	3 (75)	4 (100)	4 (100)
Male	1 (25)	0	0
Asian–Japanese heritage, *n* (%)	4 (100)	4 (100)	4 (100)
SLE disease duration, years			
Mean (SD)	14.03 (8.337)	7.41 (3.956)	6.77 (7.706)
Median (range)	15.50 (3.5–21.6)	7.30 (3.3–11.8)	3.56 (1.8–18.1)
SELENA SLEDAI score, baseline			
Median (range)	4 (2–8)	3 (2–6)	3 (0–5)
Manifestations at the time of SLE diagnosis, *n* (%)			
Malar ‘butterfly’ rash	3 (75)	4 (100)	3 (75)
Discoid rash	0	2 (50)	1 (25)
Photosensitivity	3 (75)	4 (100)	1 (25)
Oral ulcers	1 (25)	2 (50)	0
Arthritis	3 (75)	4 (100)	2 (50)
Serositis	1 (25)	0	0
Renal disorder	2 (50)	1 (25)	1 (25)
Neurologic disorder	1 (25)	0	1 (25)
Hematologic disorder	2 (50)	3 (75)	3 (75)
Immunologic disorder	4 (100)	3 (75)	3 (75)
Anti-nuclear antibody	4 (100)	4 (100)	4 (100)

Patients were able to enroll in this study regardless of sex. Only one male participated to this study and the male patient received placebo. Hence, the data generated in this study on belimumab are only applicable to Japanese females.

### Safety

A total of seven patients, three in the placebo group and two in each of the belimumab 1 mg/kg and 10 mg/kg groups, reported at least one AE regardless of relationship to study medication. Two patients in the belimumab 10 mg/kg group and one patient in the belimumab 1 mg/kg group experienced at least one AE considered to be related to study medication, but all AEs were mild in intensity (Table 2). The only AE that was considered to be drug related and which occurred in more than one subject was pharyngitis which occurred in one subject on the placebo group and one subject in the 1 mg/kg group. There were no reports of infusion-related reactions. No patient reported drug-related SAEs or AEs leading to discontinuation. One patient in the placebo group experienced a non-treatment-related SAE of severe back pain that required hospitalization and which resolved 11 days after its onset.

**Table 2.** Summary of drug-related adverse events by treatment groups.

		Belimumab	
	Placebo (*n* = 4)	1 mg/kg (*n* = 4)	10 mg/kg (*n* = 4)
Any event	2 (50)	1 (25)	2 (50)
Pharyngitis	1 (25)	1 (25)	0
Oral herpes	0	0	1 (25)
Constipation	0	0	1 (25)
Paronychia	0	1 (25)	0
Erythema	0	1 (25)	0
Rash	0	1 (25)	0
Conjunctivitis	1 (25)	0	0
Pollakiuria	1 (25)	0	0

Values are expressed as *n* (%).

No clinically significant changes in laboratory values, vital signs, or 12-Lead ECG results occurred during the study.

### Pharmacokinetics

**Figure 1.**
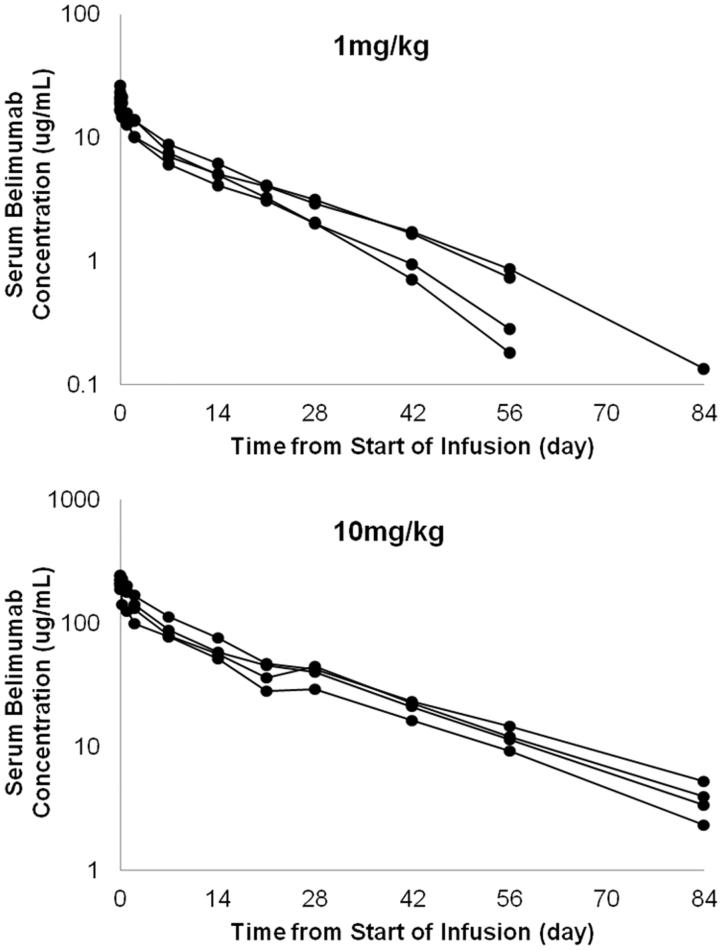
Serum concentration of belimumab after a single intravenous dose in individual patients; 1 mg/kg (*n* = 4), 10 mg/kg (*n* = 4). Serum concentrations of belimumab were determined using an electrochemiluminescence (ECL)-based assay. The lower limit of quantitation of the assay was 100 ng/mL of belimumab in 100% human serum, which was determined by multiplying 0.25 ng/mL by 400, the lowest dilution factor used in the assay.

Following single IV administration, serum belimumab concentration declined in a bi-exponential manner, with a geometric mean *t*_½,α_ of 0.644 and 0.600 days and geometric mean *t*_½,β_ of 12.395 and 15.705 day for the 1 mg/kg and 10 mg/kg groups, respectively (Figure 1 and Table 3). The geometric mean *V*_ss_ (1 mg/kg: 80.062 mL/kg and 10 mg/kg: 76.217 mL/kg) is about twice the *V*_1_ (1 mg/kg: 48.856 mL/kg and 10 mg/kg: 44.260 mL/kg). The geometric mean CL for the 10 mg/kg (3.554 mL/day/kg) group was lower than the 1 mg/kg (4.648 mL/day/kg) group, and the geometric mean MRT for the belimumab 10 mg/kg group was longer compared with the belimumab 1 mg/kg group, but the 95% CIs overlapped between the two dosing levels.

**Table 3.** Pharmacokinetics parameters by dose levels following single intravenous dose of belimumab.

Parameter	Treatment	Geometric mean	95% confidence interval
C_max_ (μg/mL)	1 mg/kg	20.241	15.265, 26.838
	10 mg/kg	222.565	193.459, 256.051
C_max_/D (kg/mL)	1 mg/kg	0.02024	0.01527, 0.02684
	10 mg/kg	0.02226	0.01935, 0.02561
AUC_0–∞_ (day·μg/mL)	1 mg/kg	215.142	165.264, 280.075
	10 mg/kg	2813.865	2104.406, 3762.504
AUC_0–∞_/D (kg·day/mL)	1 mg/kg	0.21514	0.16526, 0.28007
	10 mg/kg	0.28139	0.21044, 0.37625
*t*_½,α_ (day)	1 mg/kg	0.644	0.338, 1.230
	10 mg/kg	0.600	0.091, 3.976
*t*_½,β_ (day)	1 mg/kg	12.395	8.641, 17.778
	10 mg/kg	15.705	9.665, 25.518
CL (mL/day/kg)	1 mg/kg	4.648	3.570, 6.051
	10 mg/kg	3.554	2.658, 4.752
V_1_ (mL/kg)	1 mg/kg	48.856	36.653, 65.122
	10 mg/kg	44.260	38.827, 50.452
V_ss_ (mL/kg)	1 mg/kg	80.062	59.402, 107.907
	10 mg/kg	76.217	52.409, 110.839
MRT (day)	1 mg/kg	17.225	12.074, 24.572
	10 mg/kg	21.446	14.402, 31.935

The dose-normalized AUC_0–∞_ for the 10 mg/kg group was higher than that of the 1 mg/kg group, with an estimated ratio of 1.31 (90% CI: 1.03, 1.66). This might be due to imbalanced covariate effects between treatment groups or due to random variability due to small subject numbers (the 95% CI of the ratio overlaps with 1). The dose-normalized *C*_max_ was comparable between the two dosing levels with an estimated ratio of 1.10 (90% CI: 0.91, 1.33).

### Biomarkers and Immunogenicity

**Figure 2.**
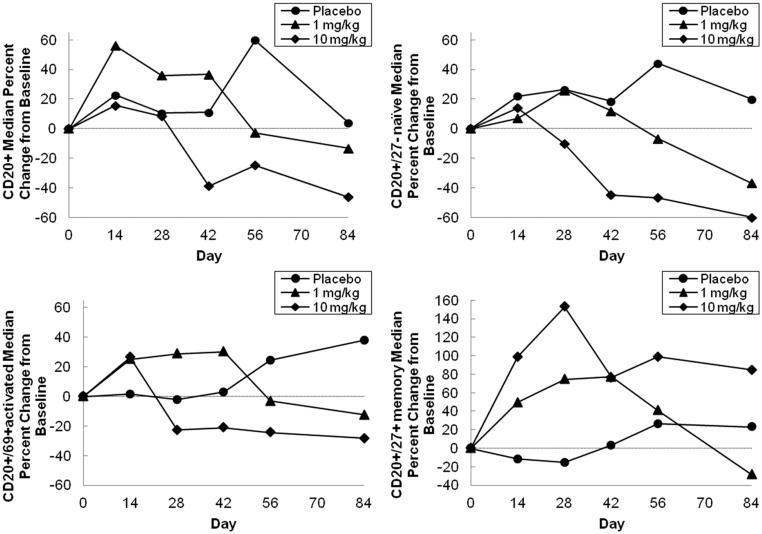
Median percentage change from baseline in CD20+, naïve, activated and memory B cells after a single intravenous dose; 1 mg/kg (*n* = 4), 10 mg/kg (*n* = 4) and placebo (*n* = 4).

Although some B lymphocyte subsets were subject to exploratory analysis to see if previously reported belimumab’s effects were reproduced, no differences were detected. In general, the percentage reduction in CD20+ B cells was greater in patients treated with belimumab 10 mg/kg than in those treated with placebo (Figure 2 and Table 4). The median percentage reduction from baseline in CD20+ B cells tended to be greater in the 10 mg/kg group than in the 1 mg/kg group. The median percentage reduction in CD20+ B cells in the 1 mg/kg group was 19.22% at day 84, while a reduction of 31.15% at day 42 and 52.49% at day 84 was reported in the 10 mg/kg group. The median percentage reductions from baseline in naïve B cells (CD20+/CD27−) in patients treated with belimumab 1 mg/kg at day 56 and day 84 were 6.80% and 37.05%; in the 10 mg/kg group a reduction of 10.46% was observed at day 28 and levels were further reduced by 46.64% and 59.95% at day 56 and day 84, respectively. In the belimumab 1 mg/kg group, the median percentage of activated B cells (CD20+/CD69+) was reduced by 3.25% on day 56 and 12.43% on day 84. In the 10 mg/kg group, the median percent reduction was 22.76% by day 28, which was sustained through day 84. There was an apparent expansion of the memory B cell (CD20+/CD27+) in patients treated with belimumab compared to placebo. The increase in memory B cells was more notable in the 10 mg/kg group compared to the 1 mg/kg group. The maximum median percentage increase of memory B cells was noted at day 42 (77.08%) in the 1 mg/kg group and at day 28 (152.88%) in the 10 mg/kg group. The changes from baseline levels of plasmacytoid cells (CD20+/CD138+), SLE B cells (CD19+/CD27^BRIGHT^/CD38^BRIGHT^), and plasma cells (CD20−/CD138+) did not show any trend over time in the treatment groups. The change from baseline in serum immunoglobulins (IgA, IgG and IgM) also did not follow a trend over time in the treatment groups. Also, the percentage change from baseline in autoantibodies (anti-dsDNA antibody and ANA) levels was not significantly different for belimumab compared with placebo.

**Table 4.** Median percent change from baseline in biomarkers following single intravenous dose of belimumab and placebo.

Parameter	Treatment	Mean baseline value	Median change from baseline (%)				
			Day 14	Day 28	Day 42	Day 56	Day 84
CD20+ (cells/µL)	Placebo	3.95	37.08	2.92	4.18	62.08	2.62
	1 mg/kg	5.95	29.12	42.12	23.10	5.03	−19.22
	10 mg/kg	10.95	1.71	−1.04	−31.15	−23.95	−52.49
CD20+/27− naïve (cells/µL)	Placebo	3.05	21.84	26.27	18.42	44.08	19.82
	1 mg/kg	4.58	7.01	25.90	11.86	−6.80	−37.05
	10 mg/kg	8.70	13.91	−10.46	−44.73	−46.64	−59.95
CD20+/69+ activated (cells/L)	Placebo	6.65	1.60	−2.21	2.98	24.43	38.05
	1 mg/kg	8.63	25.09	28.92	30.21	−3.25	−12.43
	10 mg/kg	14.93	26.46	−22.76	−21.00	−24.36	−28.26
CD20+/27+ memory (cells/µL)	Placebo	1.03	−11.81	−15.66	3.41	26.39	22.92
	1 mg/kg	1.33	49.43	74.62	77.08	40.91	−28.41
	10 mg/kg	1.30	98.61	152.88	76.39	98.72	84.72
CD20+/138+ plasmacytoid (cells/µL)	Placebo	0.08	0.0	−100.0	−100.0	0.0	−100.0
	1 mg/kg	0.08	−100.0	−100.0	−100.0	0.0	−100.0
	10 mg/kg	0.15	−100.0	0.0	−100.0	−40.0	−100.0
CD20−/138+ plasma cells (cells/µL)	Placebo	0.33	0.00	−20.00	−20.00	−30.00	50.00
	1 mg/kg	0.33	−70.83	−10.00	−37.50	−41.67	−55.00
	10 mg/kg	0.18	50.00	0.00	75.00	50.00	0.00
CD19+/27^BRIGHT^/38^BRIGHT^ SLE subset (cells/µL)	Placebo	3.65	59.25	45.72	21.43	−4.73	130.86
	1 mg/kg	2.73	−45.44	−43.85	−15.26	8.65	63.93
	10 mg/kg	0.85	−18.83	25.52	36.36	99.35	13.77
C3 (mg/dL)	Placebo	73.8	7.6	7.7	6.8	7.2	9.4
	1 mg/kg	64.3	9.5	11.7	7.4	7.0	4.7
	10 mg/kg	78.3	12.9	12.4	25.2	15.7	16.3
C4 (mg/dL)	Placebo	8.5	−5.0	5.0	15.0	10.0	0.0
	1 mg/kg	11.5	11.4	3.8	7.7	14.5	12.7
	10 mg/kg	15.3	23.0	18.1	46.9	21.9	31.3

**Figure 3.**
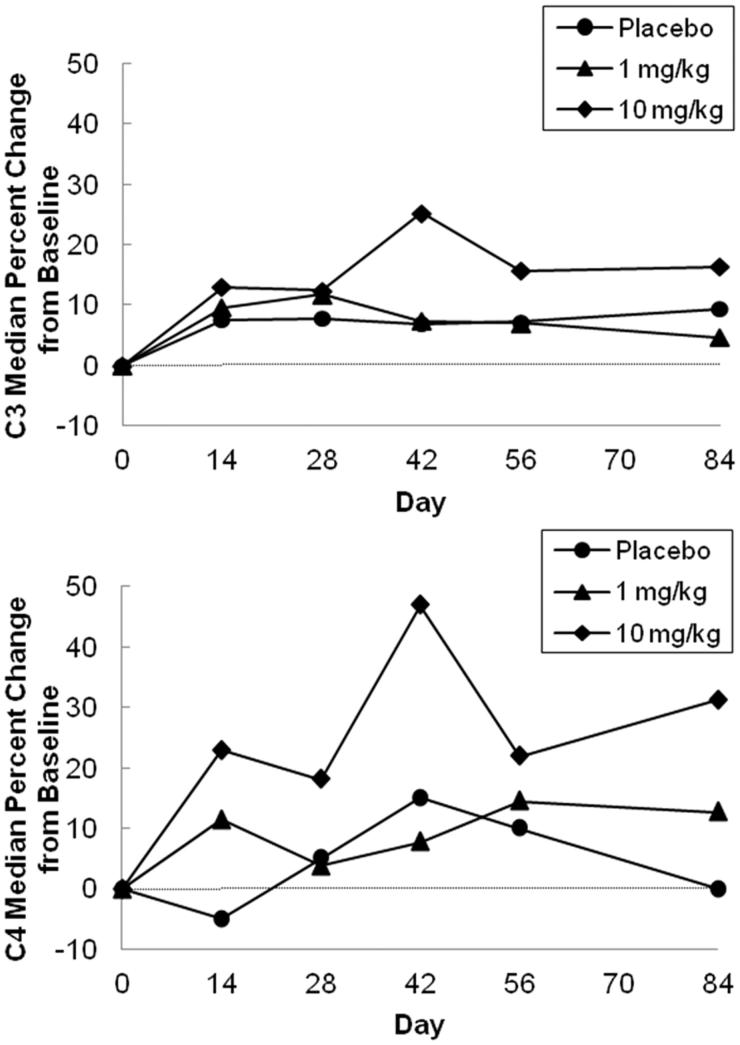
Median percentage change from baseline in complements (C3, C4) after a single intravenous dose; 1 mg/kg (*n* = 4), 10 mg/kg (*n* = 4) and placebo (*n* = 4).

The median percent increase from baseline in C3 and C4 tended to be greater in the 10 mg/kg treatment group compared to the 1 mg/kg group or placebo group (Figure 3 and Table 4).

The presence of anti-belimumab antibody was evaluated on day 1, day 14 and day 84 and all patients in each treatment group were tested negative for these antibodies.

## Discussion

This is the first report on the use of belimumab in Japanese patients. The primary objectives of this study were to evaluate the safety and tolerability of belimumab in Japanese patients with SLE, as well as to investigate the PK and biological activity of belimumab in this population.

Belimumab was generally safe and no safety concerns were found following a single intravenous administration of belimumab at both 1 mg/kg and 10 mg/kg doses in Japanese patients with SLE. No deaths, drug-related SAEs, or withdrawals due to AEs were reported; additionally, no belimumab-related laboratory abnormalities or changes in vital signs or ECG were observed. The majority of AE were classified as mild in severity. There was no relationship between the dose of belimumab and the number of incidence, severity, or type of AE reported. Considering the pivotal role of B cells in immune function, it is conceivable that a BLyS inhibitor may increase the risk of infection. However, there were no reports of moderate or severe infections. Anti-belimumab antibodies were not detected in this study. Overall, there were no unanticipated AEs in this study, although there are a number of limitations of this study, notably the small sample size and single-dose design. Thus, a full assessment of the long-term safety of belimumab in Japanese patients cannot be made.

Low serum complement concentrations and elevated anti-dsDNA antibody concentrations correlate with disease severity and can predict future flares in SLE patients10. Belimumab is a molecule expected to reduce certain B-cell subsets by blocking the activity of BLyS; BLyS receptors are expressed on most B-cell subsets including memory B cells. An agent that reduces B-cell counts might also be expected to reduce the products of B cells (i.e., immunoglobulins, including autoantibodies). A reduction in autoantibodies, in turn, could be expected to be associated with increases in complement concentrations. Thus, pharmacodynamic endpoints that have been evaluated in this study include serum immunoglobulin, autoantibody, complement (C3 and C4) and B-cell counts. The percentage reduction in CD20+ B cells tended to be greater in patients treated with belimumab than in those treated with placebo. Levels of naïve B cells (CD20+/CD27−) and activated B cells (CD20+/CD69+) tended to be decreased in the 10 mg/kg group while no change was observed in the placebo group. Therefore, administration of belimumab has been associated with decrease in B cells consistent with its mechanism of action as a BLyS inhibitor. Increases in memory B cells (CD20+/CD27+) occurred in the 10 mg/kg group. The mechanism underlying this increase in memory B cells may include release of memory B cells from disrupted lymphoid tissue, inhibition of the return of memory B cells to germinal centers, or the promotion of differentiation of naïve cells to memory B cells, although the latter may be less likely with effective binding of BLyS by belimumab11,12. The serum immunoglobulins (IgA, IgG and IgM) and autoantibodies (anti-dsDNA antibody and ANA) were not consistent and did not follow a trend in all the treatment groups. This is not unanticipated given that only a single dose of belimumab was administered, the sample size was small, and the patients with limited disease activity were included.

The single-dose pharmacokinetic profile of belimumab was approximately dose proportional and consistent with other monoclonal antibodies13. The geometric mean CL ranged from 3.55 to 4.65 mL/day/kg, which is much less than the glomerular filtration rate, indicating that renal clearance is not a major component of belimumab clearance. A half-life for terminal phase (*t*_½,β_) of 12–16 days supports dosing every 28 days in clinical program of belimumab. Although the study did not have the power to detect ethnic differences, this PK result seems to be similar to previous reports assessed non-Japanese population8,14.

The small sample size and single-dose design of this study prevent definitive conclusions regarding the safety, pharmacokinetics or pharmacodynamics of belimumab in a Japanese population being made. However, the results are not inconsistent with currently available data in non-Japanese populations.

## Conclusion

In Japanese patients with SLE, no safety concerns were found following a single intravenous administration of belimumab and the pharmacokinetic profile was consistent with that expected. Effects of belimumab on B cells, although difficult to interpret in a single-dose study, suggest pharmacodynamic effects expected of an inhibitor of BLyS. This single-dose study testing of small numbers of subjects have limitations. However, the preliminary safety, PK profile, and observed PD effects of belimumab support further evaluation of its safety and efficacy in Japanese patient with SLE and our results suggest that the treatment of Japanese SLE patients with belimumab may prove beneficial, just as it did for non-Japanese patients.

## Transparency

### Declaration of funding

This study was funded by GlaxoSmithKline and Human Genome Sciences, Inc.

### Declaration of financial/other relationships

All authors are employees of GlaxoSmithKline, K.K.

## Acknowledgments

First and foremost, the authors thank the SLE patients for participating in this study. The authors also thank the investigators who participated in the study, including Dr Y. Munakata and Dr T. Tsuru. This study was funded by GlaxoSmithKline and Human Genome Sciences, Inc.

The content reported herein is solely the responsibility of the authors.
